# Stem cells from the dental apical papilla in extracellular matrix hydrogels mitigate inflammation of microglial cells

**DOI:** 10.1038/s41598-019-50367-x

**Published:** 2019-09-30

**Authors:** Natalija Tatic, Felicity R. A. J. Rose, Anne des Rieux, Lisa J. White

**Affiliations:** 1 School of Pharmacy, University of Nottingham, Nottingham, NG7 2RD United Kingdom; 2 Université catholique de Louvain, Louvain Drug Research Institute, Advanced Drug Delivery and Biomaterials, Brussels, 1200 Belgium

**Keywords:** Mesenchymal stem cells, Stem-cell research, Inflammation

## Abstract

After spinal cord injury (SCI) chronic inflammation hampers regeneration. Influencing the local microenvironment after SCI may provide a strategy to modulate inflammation and the immune response. The objectives of this work were to determine whether bone or spinal cord derived ECM hydrogels can deliver human mesenchymal stem cells from the apical papilla (SCAP) to reduce local inflammation and provide a regenerative microenvironment. Bone hydrogels (8 and 10 mg/ml, B8 and B10) and spinal cord hydrogels (8 mg/ml, S8) supplemented with fibrin possessed a gelation rate and a storage modulus compatible with spinal cord implantation. S8 and B8 impact on the expression of anti and pro-inflammatory cytokines (*Arg1*, *Nos2*, *Tnf*) in LPS treated microglial cells were assessed using solubilised and solid hydrogel forms. S8 significantly reduced the Nos2/Arg1 ratio and solubilised B8 significantly reduced *Tnf* and increased *Arg1* whereas solid S8 and B8 did not impact inflammation in microglial cells. SCAP incorporation within ECM hydrogels did not impact upon SCAP immunoregulatory properties, with significant downregulation of *Nos2/Arg1* ratio observed for all SCAP embedded hydrogels. *Tnf* expression was reduced with SCAP embedded in B8, reflecting the gene expression observed with the innate hydrogel. Thus, ECM hydrogels are suitable vehicles to deliver SCAP due to their physical properties, preservation of SCAP viability and immunomodulatory capacity.

## Introduction

Spinal cord injury (SCI), usually caused by a car crash, fall or violence, drastically influences the quality of life and increases mortality risk by 2–5 times^1^. Spinal cord has a very low capacity for self-regeneration and cannot be fully repaired by any of the current therapies^1^.

Regardless of the type or intensity, injuries to the spinal cord create a complex deteriorating microenvironment that is not yet fully understood and is dominated by inadequate immune reaction^2^. Comprehensive studies of cellular response show that the inflammatory and proliferative phases of immune response to SCI start as expected, but difficulties occur in later transitions between the pro-inflammatory and pro-resolutive phases^2^, that is, the remodeling phase that usually follows the acute pro-inflammatory phase, does not seem to occur^3^. Thus, the inflammatory phase, initiated by microglia and led by macrophages and T-lymphocytes, remains chronically predominant^2^. This contributes to the secondary injury and has a negative effect on regeneration^3^.

Attempts to influence immune response in injured spinal cord, e.g. by macrophage depletion^4^, local or systemic delivery of anti-inflammatory drugs^5^ or immunoregulatory cytokines^6^, or adoptive transfer of “immunoregulatory” (interleukin-4 (IL4), interleukin-10 (IL10) or transforming growth factor beta (TGFβ) stimulated) macrophages^7^ have shown positive effects on regeneration in pre-clinical trials. Influencing microglia polarisation and microglia transfer have also been investigated and reviewed as therapeutic options for neuro inflammatory conditions and SCI^8^.

Influencing the local microenvironment after SCI may provide another strategy to impact upon immune response. Mesenchymal stem cells (MSC) can be used for their immunomodulatory properties^9^. However, over 90% of MSC transplanted in damaged spinal cord die or migrate away^10^. Cellular losses occurring during injection due to shear stress may be reduced by suspending MSC in an injectable hydrogel^11^. Upon injection, the gel network can protect MSC from the immediate influence of the hostile microenvironment and assist in cell localisation and retention at the injection site^12^. To stay in place, filling the irregular geometry of SCI and not be washed off by the flow of cerebrospinal fluid, the window of hydrogel gelation time would have to allow hydrogel manipulation and injection while providing a setting time fast enough to avoid hydrogel wash out (typically a couple of minutes)^13^. Hyaluronan and methyl cellulose, fibrin and various biologically derived and synthetic hydrogels carrying cells or drug loaded particles have been tested in pre-clinical phases^14^. Whilst current clinical studies have focused on the delivery of bone marrow^15^ and adipose tissue derived^16^ MSC for SCI, MSC derived from other tissues may also utilised. Stem cells from the human apical papilla (SCAP) can be easily isolated from adult immature tooth, have high proliferative and migratory potential^17^ and due to specific embryonal origin, express neural markers without neurogenic stimulation^18^. We and others have previously shown that SCAP secrete a broad pallet of neurotrophic and regenerative growth factors^19^ and possess immunoregulatory properties^20^.

Hydrogels, in addition to serving as a vehicle for local delivery of SCAP, may also actively participate in immunoregulation of the local environment. Extracellular matrix (ECM) hydrogels can be generated from almost any tissue^21^ and have been shown to contain growth factors^22^, matrix-bound nanovesicles^23^ and exposed cryptic peptides and bioactive motifs^24,25^. ECM hydrogels provide general and tissue specific cues for regeneration *in vitro* and *in vivo*^26,27^. ECM hydrogels derived from different tissues previously induced an immunoregulatory-like phenotype in unstimulated bone marrow derived macrophages *in vitro* (urinary bladder matrix and small intestine submucosa hydrogels)^28^ and displayed immunoregulatory properties *in vivo* (urinary bladder matrix, colonic mucosa and myocardial matrix hydrogels)^27,29,30^.

Our objective was thus to assess whether ECM derived hydrogels would be suitable for SCAP delivery in the scope of SCI. Since ECM hydrogels derived from both heterologous organs^31^ and homologous tissues^32^ have shown reparative effects in the central nervous system, we postulated that spinal cord or alternative tissue derived hydrogels, e.g. bone, may be suitable for SCAP delivery. In addition, we hypothesised that spinal cord or bone ECM hydrogels would present appropriate mechanical properties to be administered in a spinal cord lesion and support SCAP viability and functionality. Since immunoregulatory properties have been attributed to ECM-derived hydrogels, we postulated that this intrinsic activity, combined with SCAP, may have a synergistic impact upon mitigation of inflammation of microglial cells.

## Results

### Impact of ECM origin, concentration and composition upon mechanical properties

Gelation rate, time and final moduli can greatly influence the handling of hydrogels and their applicability *in vivo*. Rheological assessments of spinal cord-derived ECM hydrogel at 8 mg/ml (S8) and on bone-derived ECM hydrogels at 8 and 10 mg/ml (B8 and B10, respectively) were undertaken. Spinal cord derived hydrogels at 10 mg/ml were not considered due to requiring high amounts of starting material.

Once incubated at 37 °C, storage modulus (G’) of pre-gel solutions quickly became higher than loss modulus (G”), reflecting the formation of a solid hydrogel (Table 1; Supplementary Fig. 1). The origin of ECM hydrogel (bone or spinal cord), as well as the concentration in the case of bone-derived ECM hydrogel, significantly impacted hydrogel maximum storage modulus. The highest storage modulus was observed for B10 while the lowest was S8 modulus. All hydrogels had a pre-gel solution around 1 Pa and a final storage modulus lower than 1 kPa. Gelation times were also dependent on hydrogel origin but not on concentration; B8 and B10 gelled faster than S8.

**Table 1:** Hydrogel maximal moduli and gelation kinetics.

Hydrogel	Storage modulus, Mean ± SD [G’, Pa]	Loss modulus, Mean ± SD [G”, Pa]	Gelation rate, Mean ± SD [Pa/min]	Time to 50% gelation, Mean ± SD [t_50_, min]	Time to 95% gelation, Mean ± SD [t_95_, min]
S8	258 ± 17^A^	37 ± 2^A^	51 ± 9^A^	2.05 ± 0.51^A^	4.02 ± 0.67^A^
B8	499 ± 10^B^	60 ± 3^B^	96 ± 5^B^	1.36 ± 0.12^A^	3.48 ± 0.50^A^
B10	608 ± 9^C^	66 ± 4^B^	118 ± 16^B^	1.44 ± 0.25^A^	3.65 ± 0.70^A^
S/F	305 ± 28^D^	44 ± 14^B^	65 ± 4^A^	1.56 ± 0.14^A^	3.97 ± 0.26^A^

Mean ± standard error of the mean (SD), N = 3. For each parameter significance was evaluated by a One-way ANOVA followed by a Tukey post-hoc test. Conditions not linked by the same letter(s) are statistically different (p < 0.05). S8 – spinal cord ECM hydrogel 8 mg/mL, B8 – bone ECM hydrogel 8 mg/mL, B10 – bone ECM hydrogel 10 mg/mL, S/F- spinal cord ECM hydrogel 8 mg/ml/fibrin at a 75/25 ratio.

In order to increase storage modulus and to decrease gelation time of S8, fibrin was added to the pre-gel solutions at a 75/25 ratio. Addition of 25% fibrin to S8 (S/F) significantly increased the hydrogel maximum modulus and reduced the time to 50% gelation by 24% (Table 1).

### Influence of SCAP encapsulation in ECM hydrogels upon immunomodulatory properties

SCAP co-cultured with activated microglia can mitigate their LPS induced pro-inflammatory response^20^. Previously, we and others, have encapsulated SCAP in hydrogels, including alginate and hyaluronic acid^33^, ECM derived^34,35^, fibrin^36^ and gelatin methacryloyl (GelMA)^37^. Whilst these studies have investigated impact upon SCAP viability, proliferation and differentiation, to date, the impact of encapsulation upon SCAP immunomodulatory properties has not been explored.

### Impact of ECM hydrogels upon mitigation of inflammation

**Figure 1:**
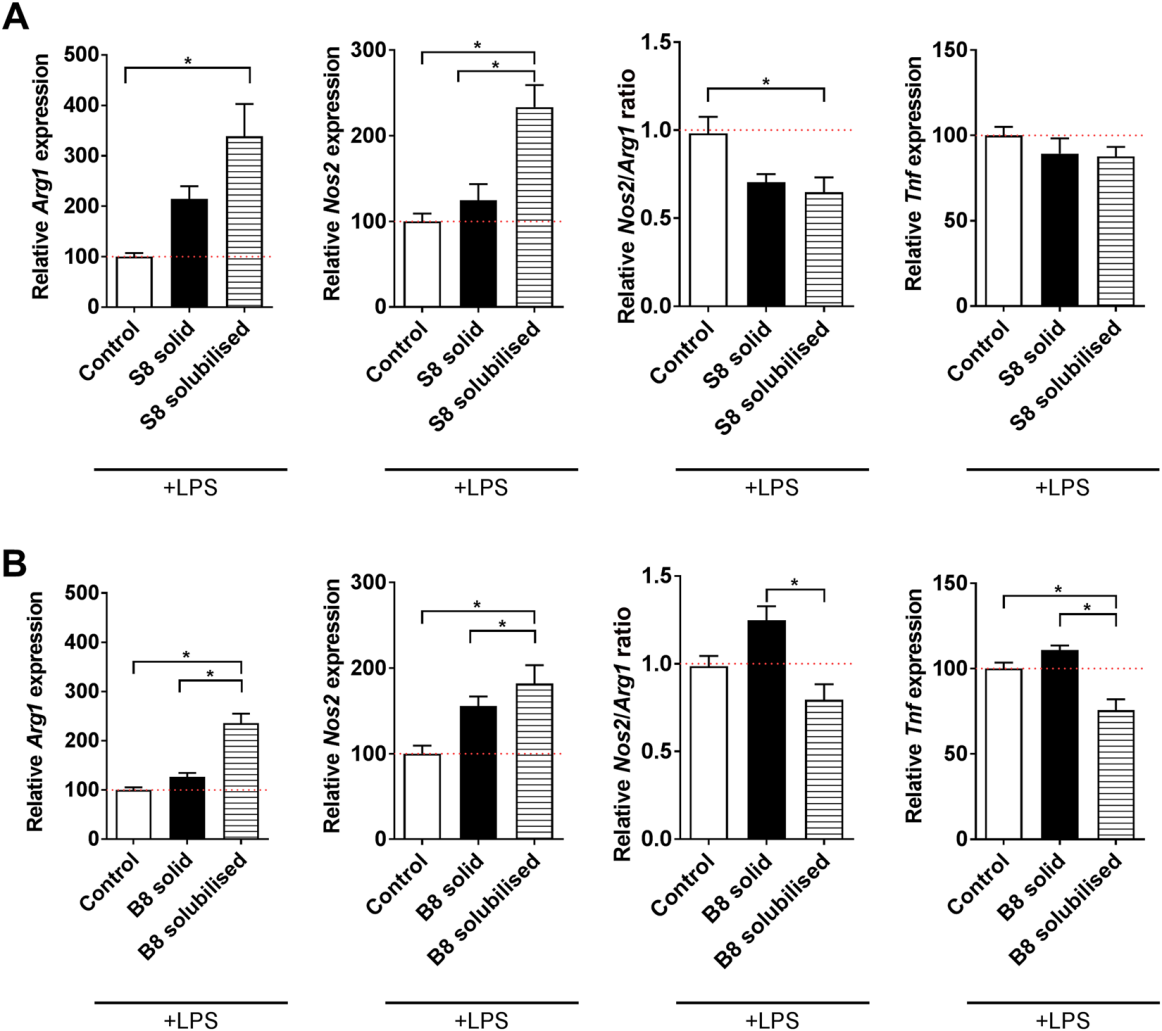
Influence of ECM hydrogels on BV2 gene expression. LPS-activated BV2 cells were treated with either solid (cast in an insert) or solubilised S8 and B8 hydrogels (directly added in culture medium) and the gene expression of tumor necrosis factor alpha (*Tnf*), inducible nitric oxide synthetase gene (*Nos2*) and arginase 1 gene (*Arg1*) was quantified by RT-qPCR. *Nos2*/*Arg1* ratio was also calculated. Results were expressed relative to the LPS-activated BV2 cells (control) set at 100% (red dotted bar). Error bars represent the standard error of the mean (N = 4, n = 3). One-way ANOVA with Tukey post-hoc test, * p < 0.05.

Immunomodulatory-like properties have been attributed to ECM hydrogels^28,29^. Hence the objective herein was to evaluate the potential impact of S8 and B8 hydrogels upon LPS-activated microglial cells (BV2 cells) by considering gene expression of markers known either for their anti-inflammatory (*arginase 1*, *Arg1*) or pro-inflammatory properties (*nitric oxide synthase Nos2*, *transforming factor alpha*, *Tnf*). The gene expression of *Arg1* and of *Nos2*, respectively considered as anti- and pro-inflammatory markers, was not significantly affected by incubation with solid S8 hydrogel (Fig. 1A; Supplementary Fig. 2A). In its solubilised form, S8 significantly increased *Arg1* and *Nos2* expression (Fig. 1A; Supplementary Fig. 2A). The resulting *Nos2/Arg1* ratios (commonly used to evaluate the immunomodulatory potential of drugs or cells)^38^ were slightly decreased, but only significantly for S8 solubilised gel. Neither forms of spinal cord ECM hydrogel impacted *Tnf* expression. B8 as a solid gel did not impact *Arg1*, *Nos2/Arg1* ratio or *Tnf* expression, although it induced an increased expression of *Nos2* (Fig. 1B; Supplementary Fig. 2B). B8 in the solubilised form significantly increased *Arg1* and *Nos2* expression (Fig. 1B; Supplementary Fig. 2B). The resulting *Nos2/Arg1* ratio and *Tnf* expression were downregulated when LPS-activated BV2 cells were incubated with solubilised B8 compared to solid B8. Neither form of B8 or S8 induced an inflammatory response in unstimulated (naïve) microglia as demonstrated by TNFα secretion (Supplementary Fig. 3).

### Impact of SCAP upon mitigation of inflammation

**Figure 2:**
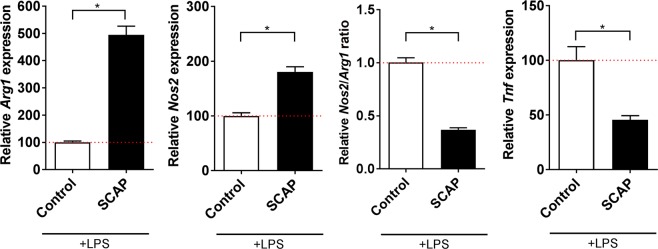
Influence of SCAP on BV2 cell inflammatory markers. SCAP seeded on inserts were co-cultured with LPS-activated BV2 cells and the gene expression of tumor necrosis factor alpha (*Tnf*), inducible nitric oxide synthetase gene (*Nos2*) and arginase 1 gene (*Arg1*) were quantified by RT-qPCR. *Nos2*/*Arg1* ratio was also calculated. Results were expressed relative to the LPS-activated BV2 cells (control) set at 100% (red dotted bar). Error bars represent the standard error of the mean (N = 4, n = 3). Unpaired t test with Welch’s correction, * p < 0.05.

SCAP can decrease *Tnf* and increase *Arg1* expression of LPS-activated microglia when co-cultured in direct contact for 48h^20^. However, their impact when co-cultured for 24 h with no direct contact (seeded on the apical side of an insert) has not been evaluated yet. In the presence of SCAP at the apical side of an insert, LPS-stimulated BV2 cells, seeded in the basolateral compartment, had significantly increased expression of *Arg1* and *Nos2* while the resulting *Nos2/Arg1* ratio and *Tnf* expression significantly decreased (Fig. 2; Supplementary Fig. 2C).

### Impact of SCAP encapsulated ECM hydrogels upon mitigation of inflammation

**Figure 3:**
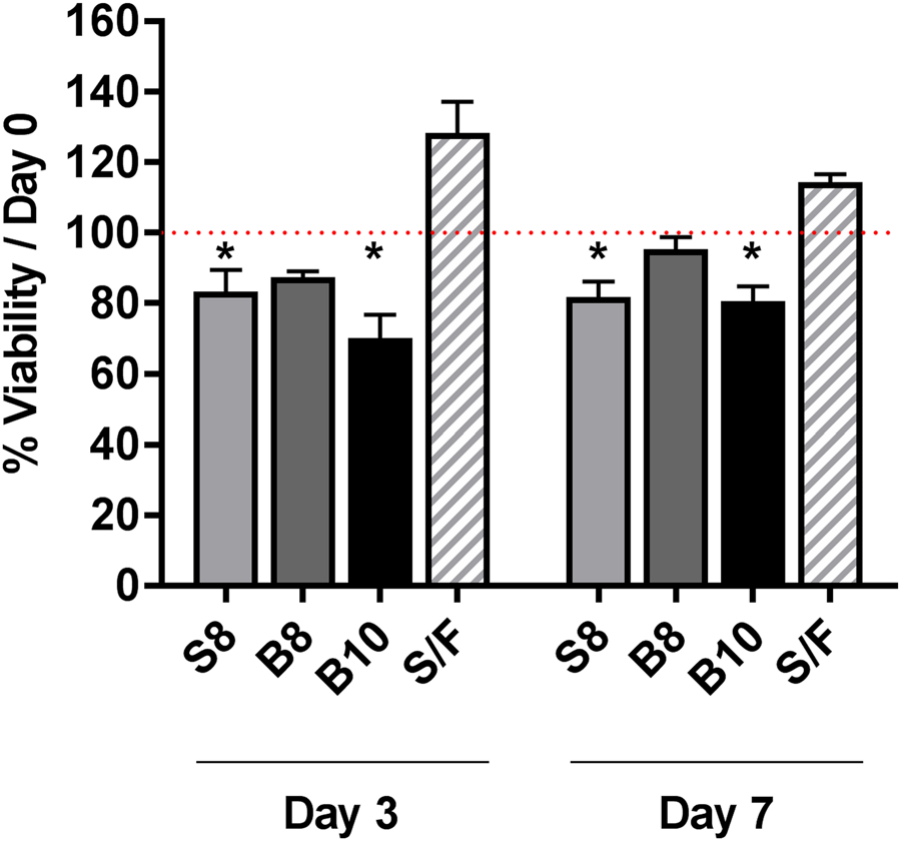
Impact of encapsulation in ECM hydrogels upon SCAP viability. SCAP viability in ECM hydrogels was assessed 3 and 7 days after incorporation in different hydrogels: spinal cord at 8 mg/ml (S8) and bone at 8 and 10 mg/mL (B8 and B10) and S8 combined with fibrin (S/F). Results were expressed relative to viability at Day 0 for each condition (red dotted bar). Error bars represent the standard error of the (N = 3, n = 3). Mixed effects model with Geisser-Greenhouse correction with Dunnett’s post hoc test, * p < 0.05.

Prior to investigating the impact of SCAP encapsulation in ECM hydrogels upon their immunomodulatory properties, SCAP viability in the hydrogels was first evaluated. SCAP were incorporated in S8, B10 and S/F hydrogels for 3 and 7 days and their metabolic activity was normalized to metabolic activity at encapsulation (day 0). SCAP viability remained above 80% 3 and 7 days after encapsulation except for SCAP encapsulated in B10 at Day 3 (70%) (Fig. 3).

**Figure 4:**
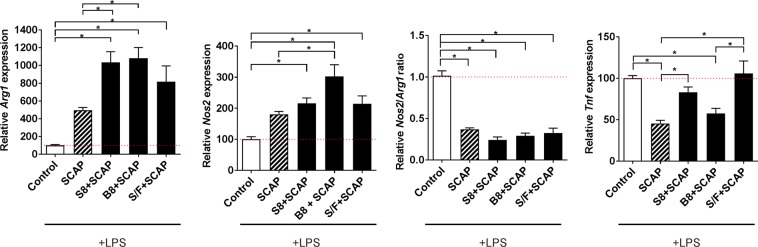
Influence of SCAP encapsulation in ECM hydrogels on their immunomodulatory properties. SCAP seeded on inserts, alone or encapsulated in S8, B8 or S/F hydrogels, were co-cultured with LPS-activated BV2 cells and the gene expression of tumor necrosis factor alpha (*Tnf*), inducible nitric oxide synthetase gene (*Nos2*) and arginase 1 gene (*Arg1*) was quantified by RT-qPCR. *Nos2*/*Arg1* ratio was also calculated. Results were expressed relative to the LPS-activated BV2 cells (control) set at 100% (red dotted bar). Error bars represent the standard error of the mean (N = 4, n = 3). One-way ANOVA with Tukey post-hoc test, * p < 0.05.

SCAP were incorporated into S8, B8 or S8 supplemented with fibrin (S/F) at a ratio of 75/25; SCAP incorporated hydrogels were located at the apical side of an insert with LPS stimulated BV2 cells seeded in the basolateral compartment. Incorporation of SCAP into all hydrogel types significantly increased *Arg1* expression relative to control (8–10-fold) (Fig. 4; Supplementary Fig. 2D). Compared to SCAP alone, incorporation of SCAP into S8 or B8 hydrogels increased *Arg1* expression by 2-fold. *Nos*2 expression was significantly increased relative to the control for all SCAP encapsulated hydrogels and significantly increased relative to SCAP alone for SCAP encapsulated in S8 and B8 hydrogels. The *Nos2/Arg1* ratio was significantly decreased relative to the control (>2-fold) when SCAP were encapsulated in all hydrogels but there was no significant difference observed when compared to SCAP alone. *Tnf* expression was significantly increased relative to SCAP alone when SCAP were encapsulated in S8 and S/F hydrogels. As a corollary to this, *Tnf* expression was significantly reduced by SCAP alone and encapsulated with B8 relative to the control.

## Discussion

Hydrogel application in SCI requires manipulation and injection of the material along with sufficiently quick gelation in the lesion^13^. The thermo-responsive nature of ECM hydrogels provides a potential role for these materials. Rather than being purely dictated by collagen self-assembly, ECM hydrogel formation is regulated by the presence of glycosaminoglycans, proteoglycans and proteins^39^. Thus, polymerization kinetics are influenced by the native biochemical profile of the original tissue and the proteins retained after decellularization^21^. We have previously shown that the biochemical profiles of bone and spinal cord derived materials are distinctly different with higher collagen and lower sulphated glycosaminoglycan (sGAG) content in bone ECM and lower collagen and higher sGAGs in spinal cord^34^. The impact of biochemical profile, i.e. of different tissue origin, was clearly shown in the rheological assessments whereby bone derived ECM gelled faster and had a higher storage moduli than spinal cord ECM. Concentration dependent gelation kinetics has previously been shown at concentrations less than 8 mg/ml for bone^40^, dermis^41^ and other tissues^21^. Herein a similar dependency was observed with storage moduli of bone hydrogels increasing with concentration, with the highest storage moduli observed at 10 mg/ml.

The low storage moduli of spinal cord derived hydrogels prompted incorporation of fibrin, as a strategy to improve mechanical properties, Fibrin has fast gelation kinetics, an easily tuneable strength and concentration and has been used previously to deliver SCAP^36^. Whilst the inclusion of fibrin increased the storage moduli and decreased time to gelation, the similarity of S/F to S8 moduli indicated that the S/F hydrogel is an interpenetrating but not interactive network and the mechanical behavior is thus predicated by the dominant entity, i.e. spinal cord ECM. Similar results have been observed for collagen fibrin co-gels where gels with higher concentrations of collagen acted as two distinct, interpenetrating, but not interacting, networks^42^.

It is essential that any tissue engineered therapy for spinal cord injury is designed with desirable mechanical characteristics in order to avoid implant-induced damage and to, maximize cellular response, regeneration and functional recovery^43^. Whilst biomechanical studies of spinal cord tissue have reported a range of modulus values (0.12–15.2 kPa)^44^, depending upon species and measurement methods, low stiffness and viscoelasticity are key parameters in selection of appropriate biomaterials. All the hydrogels assessed in this study had maximum moduli less than 1 kPa and meet these requirements.

Previously, the immunoregulatory properties of ECM hydrogels have been investigated *in vitro* with naïve (M0 state) bone marrow derived macrophages from rat and mice and those stimulated to a proinflammatory (M1-like state)^28,45^. To date, no studies have investigated mitigation of inflammation with bone or spinal cord derived ECM hydrogels nor has the influence upon microglial cells been elucidated. Microglial cells were selected due to their contribution to initiation of inflammation upon SCI2. Recent elucidation of the role of proinflammatory microglia necroptosis in driving microglial repopulation and regeneration has shown that targeting proinflammatory microglia may represent a strategy to dampen CNS inflammation^46^.

Previous *in vitro* studies have examined immunoregulatory properties of ECM hydrogels as solubilised materials, i.e. added directly to the media of stimulated cells^28^. Our desire to use ECM hydrogels to deliver SCAP led us to examine the immunoregulatory properties of both solubilised (direct contact with microglial cells) and solid forms, where the solid hydrogel was cast into a transwell insert placed above stimulated microglial cells (indirect contact). Neither form of these materials induced inflammation in unstimulated (naïve) microglial cells, similar to previous studies of solubilised ECM with unstimulated macrophages^28,45^. This is due to the similarity of ECM proteins between species^47^; both allogeneic and xenogeneic ECM hydrogels have been reported to promote  a constructive immune response and enable tissue remodeling *in vivo*^25,48^.

Only solubilised forms of ECM hydrogels mitigated LPS induced inflammation herein: addition of S8 significantly decreased *Nos2*/*Arg1* ratio and addition of B8 significantly decreased *Tnf* expression. Previous studies of ECM materials have shown that changes in the structural form of ECM may alter the presentation and conformation of ligands, thus influencing ligand-receptor interactions^49^. Additionally, different fibre conformations will influence the exposure and release of different molecules, including matricryptic peptides and matrix-bound nanovesicles, which in turn impact upon functional outcomes including cell migration and mitigation of inflammation^23,24,50^. Direct addition of solubilised S8 and B8 likely increased presentation of released molecules to stimulated microglial cell surface receptors, compared to the solid forms, resulting in mitigation of inflammation. Whilst it is not currently known why solubilised forms of S8 and B8 differentially impacted *Nos2*, *Arg1* and *Tnf* expression, this is likely predicated by the different biochemical profiles of each tissue as tissue specific induced expression of anti-inflammatory markers has previously been observed^28^.

The immunoregulatory properties of SCAP have previously been observed^20^ and were further confirmed herein as SCAP mitigated LPS-induced inflammation of microglial cells with a significant decrease in both *Nos2/Arg1* ratio and *Tnf e*xpression. Encapsulation in ECM hydrogels did not impede SCAP mitigation of LPS-induced inflammation as demonstrated by a significant downregulation of *Nos2/Arg1* ratio observed with all hydrogels. However, encapsulation of SCAP in S8 and S/F hydrogels led to increased *Tnf* expression compared to SCAP alone. Encapsulation of SCAP in B8 significantly reduced *Tnf* expression relative to control and to a similar level of SCAP alone.

The different impacts upon gene expression from SCAP embedded in different source tissue hydrogels mirror the impacts observed with hydrogels alone. S8 alone reduced the *Nos2/Arg1* ratio and whilst not significant, the addition of SCAP to S8 tended to further decrease the *Nos2/Arg1* ratio compared to SCAP alone. Similarly, the decrease in *Tnf* expression was only observed with B8, SCAP alone and SCAP in B8. It appears that the immunoregulatory properties of SCAP embedded hydrogels reflects not only the innate properties of SCAP but also the specific immunoregulatory properties of the individual ECM hydrogels.

Further investigation of the biochemical profiles of the tissue specific hydrogels is required in order to clearly elucidate the mechanisms by which SCAP and hydrogels in combination modulate inflammation. We intend to use omics analyses to explore the immediate impact of ECM hydrogel composition upon SCAP secretome and downstream regulation of inflammation.

In conclusion, bone hydrogels and spinal cord hydrogels supplemented with fibrin had a gelation rate and storage modulus suitable for delivery of SCAP to SCI and all hydrogels supported SCAP viability. Solubilised S8 and B8 modulated inflammation of LPS stimulated microglia with differential impacts upon gene expression: S8 significantly reduced *Nos2/Arg1* ratio and solubilised B8 significantly reduced *Tnf* expression. Encapsulation of SCAP within ECM hydrogels did not impede SCAP mitigation of LPS-induced inflammation, with significant downregulation of *Nos2/Arg1* ratio observed with all hydrogels. SCAP embedded ECM hydrogels reflected both the immunoregulatory properties of SCAP and of the individual hydrogel with B8, alone or with SCAP significantly reducing *Tnf* expression. Thus, ECM hydrogels are suitable vehicles to deliver SCAP for SCI due to their physical properties, preservation of SCAP viability and immunomodulatory capacity.

## Materials and Methods

### Chemicals and reagents

RNeasy Mini Kit was from Qiagen (Manchester, UK). Sodium Ethylenediaminetetracetate (EDTA) was from AppliChem Panreac (Darmstadt, Germany). Fibrinogen and Thrombin were from Baxter Innovations GmbH (Vienna, Austria). Forty-eight-well cell-repellent plates and tissue culture inserts (pore size 1 µm) were from Greiner Bio-One (Stonehouse, UK). Triton X-100, sodium deoxycholate, peracetic acid concentrated, sterile apyrogenic 10x phosphate buffer saline (PBS), pepsin, sodium hydroxide, Minimum Essential Medium (MEM), foetal bovine serum (FBS) and lipopolysaccharide (LPS) from *Escherichia coli* 0111: B4 were from Sigma-Aldrich (Poole, UK). All other reagents were purchased from Thermo Fisher Scientific (Loughborough, UK).

### Tissue decellularization

Fresh bovine tibiae were harvested from cattle aged 12–24 months, slaughtered by an EU certified butcher (J. Broomhall Ltd, Eastington, UK). Bones were received in segmented form and were separated into cancellous and cortical groups, with the cancellous group used in this study. Fresh porcine spinal cords were obtained from market weight animals at a local slaughterhouse (Abattoird’Anderlecht, Brussels, Belgium). Female and male animals were used indiscriminately. Spinal cords and cancellous tibias were fragmented and processed following previously described protocols^34,40^. The obtained ECM were lyophilised and stored at −20 °C for further use.

### Preparation of ECM hydrogels

Obtained ECM were ground to form powders and digested with 1 mg/mL pepsin in 0.01 N hydrochloric acid for 48 h for spinal cord ECM and 72 h for bone ECM. Final solutions of 10 mg spinal cord ECM/mL and 10 and 12.5 mg bone ECM/mL were obtained, as previously described^34^ and stored at −20 °C. Prior to use, ECM digests were thawed, neutralised with 10x PBS and 0.01 N sodium hydroxide and diluted to desired concentrations (8 or 10 mg/ml) using PBS, at 4 °C, as described previously^34^. Obtained pre-gels were incubated for 20 minutes at 37 °C to form gels.

Fibrinogen and thrombin were reconstituted following manufacturer instruction, diluted to 50 mg/mL and 50 IU/mL, respectively, and used in equal final volumes as described previously^36^. Hydrogel combination with fibrin (75:25 ECM:Fibrin ratio) was made by mixing pre-gel solution with thrombin, dispensing the mixture and then adding fibrinogen to the mixture *in situ* before the incubation.

### Mechanical characterisation of ECM hydrogels

Mechanical characterisation was performed on a rheometer (Anton Paar Physica MCR 301, Austria) according to American Society for Testing and Materials (ASTM) F2900-11 standard using 200 µL of material, 25 mm parallel plates, 0.2 mm gap and a Peltier plate to control the temperature within 0.1 °C. Pre-gel solutions were placed on a pre-cooled plate (4 °C). For hydrogels containing fibrin, the pre-gel solution and thrombin was mixed with fibrinogen (12.5% of the total volume) directly on the plate. Equal distribution of material between the plates was ensured by rotating the upper plate (shear rate 1000 s^−1^) for 20 s at 4 °C. Then an oscillatory time sweep test (1% oscillatory strain, 1 rad/s angular frequency) was conducted to follow the gelation kinetics for 20 min. The temperature rose from 4 to 37 °C, at 0.5 °C/s, the fastest steady heating rate. The rest of the analysis was conducted at 37 °C. Frequency sweep test (1% strain, 0.01–100 rad/s angular frequency) followed by amplitude sweep test (0.01–200% strain, 1 rad/s angular frequency) were conducted in sequence at 37 °C. Analysis was performed in triplicate.

### SCAP culture

Human SCAP (RP89 cell line) were kindly provided by Prof Diogenes (San Antonio University, USA). SCAP were isolated from a healthy donor wisdom tooth after informed consent was provided; the study was approved by the Institutional Review Board of the University of Texas Health Science Center at San Antonio, San Antonio, TX. A SCAP cell line (RP89) was established and fully characterised as described by Ruparel *et al*^51^. Human SCAP (RP89 cells)^51^ were used between passages 5 and 10. SCAP were grown at 37 °C in 5% CO_2_ in growth medium composed of Minimum Essential Medium supplemented by 10% foetal bovine serum, 1% of L-glutamine and 1% of penicillin and streptomycin. SCAP were passaged upon reaching about 80% confluence using StemPro Accutase^®^.

### SCAP viability in ECM hydrogel combinations

SCAP viability in individual hydrogels was determined using PrestoBlue cell viability agent following supplier instructions. SCAP were suspended in pre-gel solutions at 1 × 10^6^ cells/mL and 150 µL was cast in 48-well tissue culture plates at 4 °C. Plates were then incubated at 37 °C for 20 minutes for gelation to occur. Growth medium was added on top and changed every other day up to the desired time point. At each time point, growth medium was removed and cells were incubated for 5 hours in 10x PrestoBlue dilution in medium. Cell viability was evaluated by sampling 100 µL from the solution above the gel, measuring sample fluorescence at 560 and 590 nm (emission and excitation) following the manufacturer’s protocol. Cells were incubated for 3 hours with the PrestoBlue reagent. Cell viability was expressed as a percentage of SCAP viability relative to Day 0. Cell viability experiments were performed in triplicates (N = 3, n = 3). Each type of hydrogel was used alone as negative control and the absorbance of the PrestoBlue reagent solution was subtracted from the obtained values for corresponding hydrogel with cells.

### Anti-inflammatory properties of ECM hydrogels and SCAP

#### BV2 cell culture

Mouse brain microglia cells (BV2 cells) were used between passages 21 and 31 (ATCC^®^ CRL-2469^TM^). BV2 cells were grown at 37 °C in 5% CO2 in Dulbecco’s Modified Eagle Medium GlutaMAX with 4.5 g/L D-glucose and without pyruvates supplemented by 10% foetal bovine serum and 1% of penicillin and streptomycin. BV2 cells were passaged upon reaching over 90% confluence using StemPro Accutase^®^ BV2 cells  stimulated by LPS are widely used as a model of neural inflammation^52^. Upon stimulation by LPS, 90% of the markers expressed by BV2 reflect similar trends to that observed in LPS stimulated microglia^53^.

#### BV2 stimulation and treatment

BV2 cells (1.5 × 10^5^ cells/well) were seeded in 12-well tissue culture plates using SCAP growth medium. After 16 h, the medium was either replaced with SCAP growth medium containing 100 ng/mL LPS (LPS from Escherichia coli 0111: B4, Sigma- Aldrich) for the LPS-stimulated condition or with fresh SCAP growth medium for the control condition. Hydrogel and/or cell treatments were added 2 h after LPS stimulation of BV2 cells and cultured for a further 24 h. S8 or B8 were added either as solubilised pre-gel solutions (0.5 mg/mL final concentration) directly into BV2 cell medium or as solid hydrogels in the apical compartment of cell culture inserts (pore size 1 µm) cast above BV2 cells (in the basolateral compartment). SCAP were either directly seeded on the insert membrane or incorporated in solid hydrogels (1.5 × 10^5^ SCAP/gel). Cells were stored in RLT buffer (RNeasy Mini Kit) at −80 °C until further analysis. Each experiment was performed in triplicate (N = 3, n = 3). Controls were non-activated BV2 cells (M0) and non-treated activated BV2 cells (Control).

#### Tumour necrosis factor alpha (TNFα) quantification

The concentration of murine TNFα in cell culture medium was determined by a commercially available enzyme-linked immunosorbent assay (ELISA) kit (detection limit 5.0 pg/mL) according to manufacturer’s instructions (Invitrogen^TM^).

#### BV2 gene expression analysis

Total RNA was extracted using RNA Isolation Kit (RNeasy Mini Kit) following the manufacturer’s instructions. Qualitative and quantitative analysis of RNA was performed by spectrophotometry (NanoDrop ND- 1000 Spectrophotometer, Labtech, Heathfield, UK). Given the major role that nitrogen monoxide (NO) has in the neurotoxic effects of microglia the following genes were chosen: pro-inflammatory *Nos2* coding inducible nitric oxide synthetase that produces NO and anti-inflammatory *Arg1* coding arginase 1 enzyme that spends the NO and pro-inflammatory *Tnf* coding TNFα^53^. Proinflammatory (*Tnf*-Mm00443258_m1 and inducible nitric oxide synthetase (*Nos2*) -Mm00440502_m1) and anti-inflammatory (liver arginase (*Arg1*)- Mm00475988_m1) gene expression was determined relative to ribosomal protein L19 gene (*Rpl19*- Mm02601633_g1) according to the TaqMan gene expression assay protocol using TaqMan primers and probes.

### Statistical analysis

Statistical tests were performed using Prism (GraphPad Prism 7.02 software, CA, USA). Results are expressed as mean +/− SEM and were considered significant if p < 0.05. In figure captions, detailed information about number of replicates, type of statistic test and post-test can be found for each experiment.

## Supplementary information

Supplementary Data

## Data Availability

All data generated or analysed during this study are included in this published article and its Supplementary Information files.
