# Supplementary material for: Stem cells from the dental apical papilla in extracellular matrix hydrogels mitigate inflammation of microglial cells
Source: Sci Rep. 2019 Sep 30;9:14015. doi: 10.1038/s41598-019-50367-x (PMC6768850; doi:10.1038/s41598-019-50367-x)
Supplement: Supplementary file 1 — Supplementary Data [file 41598_2019_50367_MOESM1_ESM.docx]

**Stem cells from the dental apical papilla in extracellular matrix hydrogels mitigate inflammation of microglial cells**

Natalija Tatic^1,2^, Felicity R. A. J. Rose^1^, Anne des Rieux^2*^, Lisa J. White^1*¥^

Affiliations:

^1^School of Pharmacy, University of Nottingham, NG7 2RD, United Kingdom

^2^Advanced Drug Delivery and Biomaterials, Louvain Drug Research Institute, UCLouvain, Université Catholique de Louvain, 1200, Belgium

*AdR and LJW contributed equally

^¥^Correspondence should be addressed to: lisa.white@nottingham.ac.uk

**SUPPLEMENTARY INFORMATION**


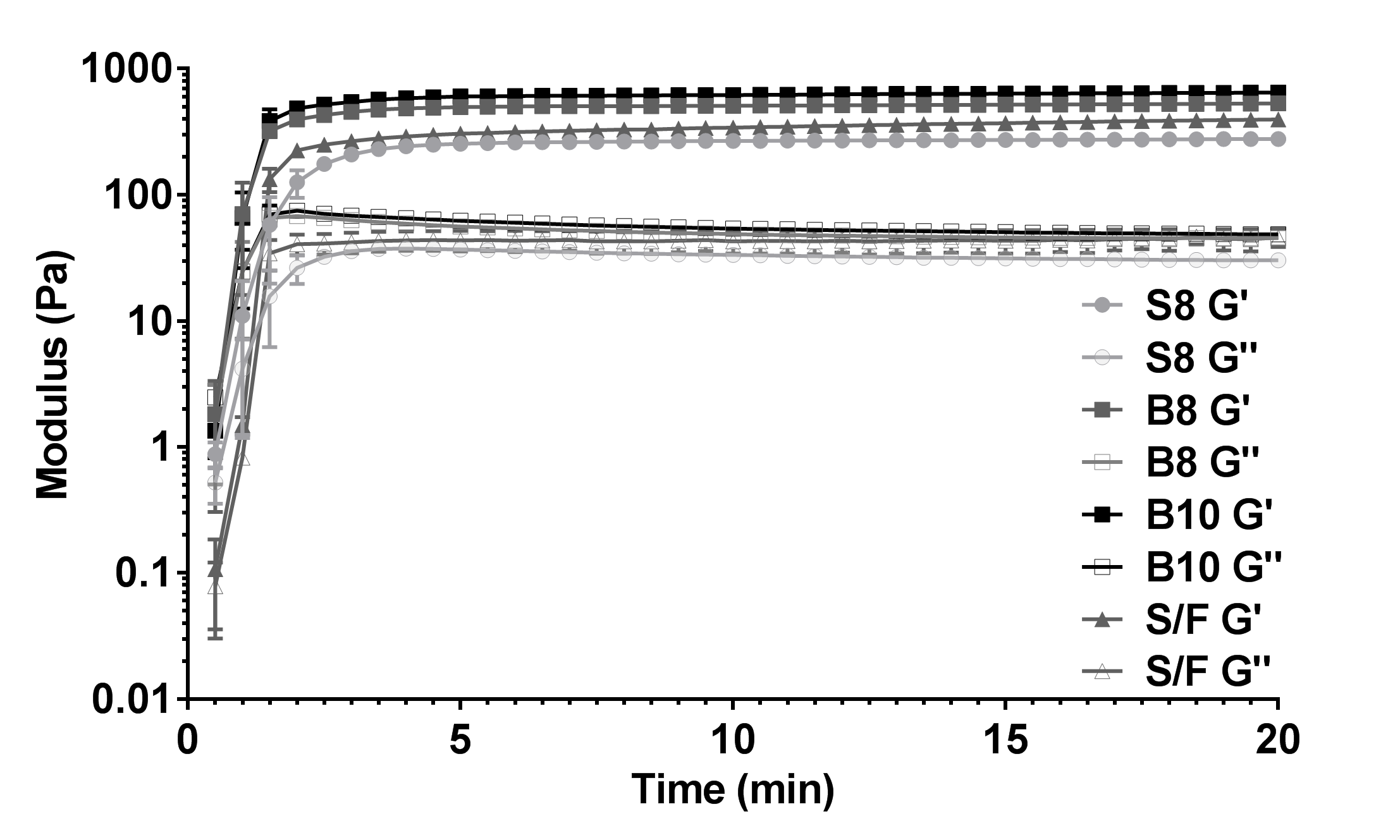


Supplementary Figure 1: Rheological characterisation of ECM hydrogels. Oscillatory time sweep curves of mean storage (G’) and mean loss (G”) moduli of different concentrations (8 and 10 mg/mL) of spinal cord (S8) and bone (B8, B10) derived hydrogels and S8 combined with fibrin (S/F). Error bars represent the standard error of the mean for three replicates (n=3).


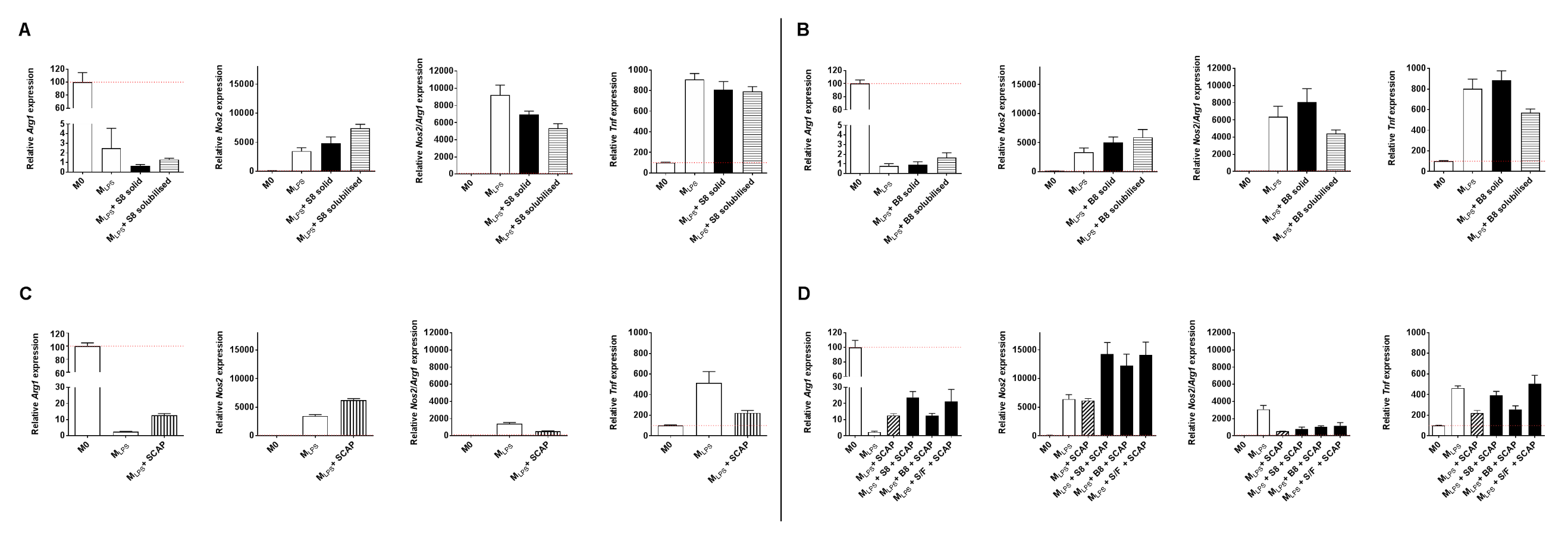


Supplementary Figure 2 Influence of ECM, SCAP or SCAP encapsulated in ECM hydrogels on BV2 cells. LPS-activated BV2 cells were treated with either solid or solubilised S8 hydrogels (A); solid or solubilised B8 hydrogels (B); SCAP seeded on inserts (C) or SCAP seeded on inserts, alone or encapsulated in S8, B8 or S/F hydrogels (D). The gene expression of tumor necrosis factor alpha (*Tnf)*, inducible nitric oxide synthetase gene (*Nos2*) and arginase 1 gene (*Arg1*) was quantified by RT-qPCR. *Nos2/Arg1* ratio was also calculated. Results were expressed relative to the non-stimulated BV2 cells (M0) set at 100% where appropriate (red dotted bar) and LPS-activated BV2 cells are also shown.


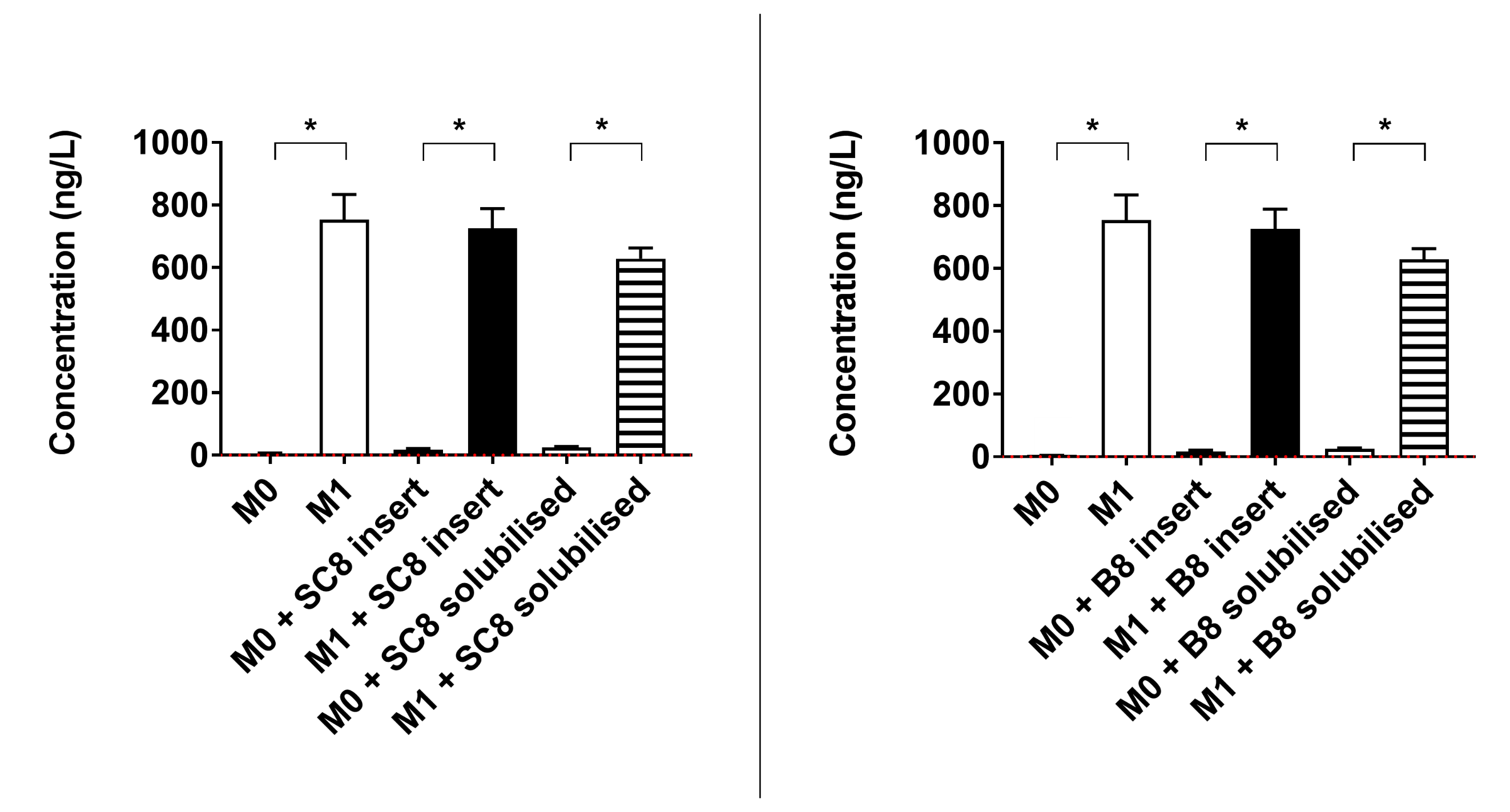


Supplementary Figure 3: Influence of ECM hydrogels on BV2 TNFα secretion. Non-stimulated (M0) and LPS-activated (M1) BV2 cells were treated with either solid (cast in an insert) or solubilised S8 and B8 hydrogels and secretion of TNFα was quantified by ELISA. Error bars represent the standard error of the mean (N=3, n=3). One-way ANOVA with Tukey post-hoc test, * p < 0.05.
